# Medical Certificates in Lunacy

**Published:** 1863-01

**Authors:** 


### Art. XII.—MEDICAL CERTIFICATES IN LUNACY.

The recent trial of Hall *v.* Semple has brought again under close consideration and bitterly angry discussion the power which the statute delegates to two qualified medical men, to certify, with a view to the imposition of legal restraint, either in or outside an asylum, persons alleged to be insane, and, therefore, fit subjects for confinement. This is no new subject to us. We have been long keenly alive to its importance, as well as to the generally entertained opinion in regard to what *primâ facie* appears to be a most arbitrary power, which the law invests in the hands of any two medical practitioners. It is useless, in the face of strongly expressed public opinion, to contend that the authority thus placed in the hands of two qualified medical men is rarely if ever abused. Twenty years' experience in matters of lunacy proves to our satisfaction, that as a general rule, to which the exceptions are most rare, the members of our honourable but much maligned profession are scrupulously and conscientiously careful in investigating cases of lunacy, previously to committing to writing the expression of an opinion authorizing the exercise of legal control and supervision. The fact of the case of Hall *v.* Semple being the first of the kind tried in a British court of law, clearly establishes the truth of the above assertion. Into the details of this painful case it is not our intention now to enter, neither do we desire to put ourselves forward as Dr. Semple's unqualified apologists. That he acted in perfect good faith with regard to his certificate of Mr. Hall's lunacy there can be but one opinion among those acquainted with the facts of the case, and who appreciate Dr. Semple's character for honour, honesty, and integrity. It would have been better if he had exercised more caution previously to writing his certificate. But we can well understand the feelings of Dr. Semple when called upon to act in a case of delusions similar to those under which Mr. Hall was supposed to labour. Dr. Semple was, no doubt, fully alive to the dangerous character of such morbid mental impressions; he knew, what every experienced medical man is cognizant of, that in the whole category of insane delusions, the one pre-eminently above all others fraught with most mischief, often leading to the sacrifice of life, is that associated with ideas of unfaithfulness on the part of the wife. We can therefore well understand Dr. Semple's great anxiety to protect the wife against the commission of any overt act of violence which the husband might be driven to by the alleged delusive idea which was represented to have taken possession of his mind. If Dr. Semple had entirely ignored the idea of lunacy in Mr. Hall's case, and had said to the wife (whose truthfulness he had no right to question), "Madam, I cannot see your husband until he voluntarily permits me to go into his presence, or I make full inquiry into your own character," the afflicted wife would, in all probability, respond, "My husband is insane; he has delusions with respect to myself. He says I am a drunken woman; that I am ruining his business, and that I am guilty of acts of infidelity. Acting on these false and insane ideas, he threatens my life, and has frequently committed acts of brutal violence upon my person." Dr. Semple, proceeding with extreme caution, being keenly alive to his personal responsibilities, recognising as hanging *in terrorem* over his head actions at law, and a heavy bill of costs to pay, hesitates to obey the wife's summons, and again informs her that he cannot comply with her wishes to see and examine the state of her husband's mind; that he will not and dare not force himself into his presence. Failing to obtain protection against the insane acts of the husband, the wife returns to her unhappy home. The infuriated lunatic, oblivious of all healthy affection for the wife, driven to desperation by the one dreadful hallucination which absorbs all his thoughts and embitters his existence, sacrifices her life. The man is arraigned, and tried for the murder of his wife. Insanity is urged in his defence. The whole facts of the case come before the Central Criminal Court. Dr. Semple is called upon as a most material medical witness. Will there be any difficulty in predicating what would be the animadversions of the counsel, or the commentary of the judge, if Dr. Semple were compelled to say that he had been consulted by the wife previously to her death, under the impression that her husband was insane, being under the dominant influence of a delusion that so frequently leads to the commission of murder, and had refused to see him until he had made particular inquiries of a number of his neighbours as to the degree of credence he was justified in placing in the *ex-parte* representation of the wife, or that he did not consider himself justified in forcing himself into the presence of the alleged lunatic in opposition to his wishes? How indignantly would counsel censure Dr. Semple for his cruel professional negligence ! It would be useless for him to say he could not believe the uusupported statement of the wfie, or that he refused to visit her husband, unless he voluntarily consented to submit to the proposed examination. We say thus much in vindication of Dr. Semple. In the various terrible onslaughts which have been made upon him, we do not think this right view of his responsible professional position has been taken. It is clear he acted *bonâ fide* in the matter, and the apparent insufficiency of the preliminary inquiries he made before signing the certificate, may be attributed to his great anxiety to protect the wife from what he considered to be the dangerous delusions of the husband. Surely it is not fair to Dr. Semple to ignore this reasonable explanation of his conduct. Do not let us be misunderstood. We repeat what we have again and again urged in the pages of this Journal, that medical men cannot be too guarded in all examinations of persons alleged to be insane, previously to committing themselves to a written opinion of insanity. In cases similar to Mr. Hall's, it is absolutely necessary, when the alleged condition of mental aberration is not distinctly *primâ facie*, to be particularly guarded in the medical investigations of the case, and not to assume the accuracy of alleged facts without a stringent examination into their character. Dr. Semple did, undoubtedly, make what he considered, according to his judgment, to be the necessary preliminary investigations, but it is clear if he had seen the various members of Mr. Hall's family, and they had stated to him what they subsequently deposed to on oath in the Court of Queen's Bench, he never would have incurred the heavy responsibility of signing the certificate that consigned Mr. Hall to a lunatic asylum. This case will be a lesson, we hope, to members of the medical profession. They should consider it as a beacon a-head; a kind of medico-psychological Goodwin Sands, upon which their vessel may be grounded and fatally wrecked. If a mistake of this nature be committed, even with an intense desire on the part of the medical profession to do what is right, we can expect no quarter either from the gentlemen of the bar or the newspaper critics that are so eager to scarify and crucify that section of our profession whose misfortune it is to have to deal with lunacy cases. "Misfortune," we repeat. Why should it be so ? Surely there is not a higher or more philosophical branch of inquiry than that which relates to the human mind in its abnormal manifestations ; and yet the men who pursue these inquiries, and are devoting the best energies of their lives to the alleviation of the severest type of human suffering, are constantly exposed to the most unrighteous abuse, and are viewed as occupied in the pursuit of a degraded calling.

To return to the vexed question of medical certificates. Should the law relating to this subject be altered? and what revision in the statute would meet the public approbation? In 1858 we brought this matter before the profession, and in reference to the points now under consideration we thus expressed our opinion:—" There always has been a great outcry against the power which the law places in the hands of two qualified medical practitioners. *Primâ facie* there are undoubtedly grave objections to this clause. If we always could guarantee the respectability, the intelligence, and the practical experience of the members of the medical profession called upon to certify to the mental condition of a person prior to his being placed under legal restraint, no. possible objection could be raised to the law as it at present exists; but unfortunately it does occasionally happen that very incompetent men are called in to certify, and by doing so without sufficient ground or reason, serious odium is brought upon all persons associated with asylums for the treatment of the insane. The force of public opinion is beyond all doubt against this part of the legislative enactment; and we had better therefore, with a good grace, bow with submission to the *vox populi,* and consent in this particular to some modification of the law.

" It is urged by those who object to the power delegated by the act to two qualified medical practitioners, that it is not right, because the insane are no longer treated with great harshness, cruelty, and brutality, not being chained, whipped, and tortured as they were in the barbarous times long since passed away, that therefore any man should be subjected to restraint in an asylum conducted by men of unquestionable respectability, and in conformity with the most enlightened and humane principles of treatment, on the simple written testimony of two apothecaries, who may never have had any previous experience in the investigation of cases of imputed insanity. I consider it to be our bounden, our sacred duty, by some amendment of the law, to fully satisfy the public mind that every safe and proper precaution has been taken to thoroughly examine, in the minutest particular, the mental condition of every person represented to be of unsound mind, and a fit subject to be deprived for a time of his civil rights.

" From what I know of the existing state of public feeling upon this point, I feel assured that it would be unwise on the part of those personally interested in the confinement of the insane to offer any opposition to an amendment of the law relative to medical certificates. It has been proposed, with a view of obviating this difficulty, and bringing the Act of Parliament more in harmony with the force of public opinion, that a *quasi*-judicial investigation should be instituted in every case previously to confinement. The Law Amendment Society suggested that an inquiry, similar to a commission of lunacy, should take place prior to the exercise of restraint, and advised that no person should be removed to a lunatic asylum who had not been pronounced by a competent jury to be of unsound mind, and in a condition to justify this mode of treatment. I am sure I need not occupy your valuable time in pointing out the absurdity and impracticability of this suggestion. With a view to the constitution of a less exceptional and incorruptible tribunal, it is proposed that a *Court of Commissioners of Insanity* should be formed, consisting of six or seven experienced men of high repute, who should be empowered to decide on the necessity of restraint in every case of alleged insanity. This Court is to be delegated with the authority of examining medical men upon oath, and if necessary, seeing the person presumed to be insane. Were such a preliminary course necessary in order legally to confine the insane, I very much fear it would greatly add to the statistics of chronic and incurable insanity. I think it would be most unwise, injudicious, and impolitic to throw any very stringent or vexatious impediments or obstructions in the way of confining the insane. Sensible, as all must be who practise in this department of medicine, of the enormous curative advantages which result from the immediate removal of cases of acute insanity from the associations of home to a well-organized and humanely conducted institution for the treatment of morbid conditions of mind, it behoves us to sanction no alteration in the law that would obviously and seriously interfere with this important principle of treatment. What is the alteration, it may be asked, that I would suggest to meet the difficulties referred to? The law at present requires two qualified medical men should personally, and apart from any other practitioner, examine the patient, and certify to the fact of insanity, specifying at the same time the facts upon which they have based their opinion.

"It has been proposed, with a view to altering the law and satisfying the requirements of public opinion, that instead of two medical certificates, *three*, or even *four*, should be required in every case previously to the imposition of restraint, and that at least *one* or two of the certificates should bear the signatures of physicians of high character and of known repute and experience. I am bound, however, to confess, from what I know of the state of public feeling on this point, that even this great concession to the popular outcry would not be satisfactory. To meet the objections raised, and to place this matter beyond all further cavil and dispute, I would suggest the appointment of educated, respectable, and experienced practitioners, delegated with *quasi*-judicial and magisterial functions, to be summoned for the purpose of countersigning the certificates of the medical men, thus sanctioning, if they thought proper, the proposed measure of confinement. These Inspectors of Lunacy, or medico-legal jurists, might be appointed to preside over certain districts in the metropolis as well as in the provinces. Being unconnected with and unknown to the relations and friends of the patient, and strictly independent of the medical men called in by the family to certify to the fact of insanity, I feel assured that the signatures of gentlemen holding such independent official appointments would relieve the public mind of all undue anxiety relative to the unjust confinement of persons alleged to be insane. I think, also, it would be considered as a boon to the medical men certifying to the fact of insanity, as well as to the family of the invalid, by placing their conduct in the matter beyond all doubt and suspicion."

It has been proposed that medical certificates in lunacy should assume the form of affidavits, and that no document of the kind should be legal, which was not, after being sworn to by the medical man, countersigned by a magistrate or justice of the peace. If any alteration of this kind is made in the law, we hope that a medical man certifying to the existence of insanity will be protected against vexatious actions at law.

We make these suggestions because medical men occupying high positions are beginning already to shrink from the grave responsibility of certifying in cases of insanity. If the contagion of their example should spread through the profession, the consequences to the public will be seriously inconvenient and to those unhappily afflicted with insanity very disastrous. With a view to their own protection, medical men will, we fear, require for the future, previously to signing certificates of lunacy, an indemnity against all pecuniary liability, should they be required to defend themselves in a court of law. We make no excuse for wilful negligence or insufficient inquiry on the part of the medical man assuming the grave and solemn responsibility of consigning fellow-creatures as insane to a lunatic asylum. He is bound to fully satisfy his own mind, after the most careful examination, as to the existence of mental aberration; but having done this (even if he should have committed an error of judgment,) the law is bound to protect him in the discharge of an onerous and at all times a most distasteful professional duty. It will be difficult for medical men to steer clear of actions at law, unless the phrases adopted by Mr. Justice Crompton, viz., "insufficient inquiry," "negligence," are clearly and definitely explained.

We much fear that this inquiry will be productive of mischief to the insane themselves, and it is on this account that we lament the necessity that existed for the legal proceedings against Dr. Semple.

If the threatened bill of exceptions should be presented to a higher tribunal, we hope that this important matter will not be lost sight of. The judges might be called upon to give a legal definition of the words "negligence," and "insufficient inquiry," for the guidance and safe protection of medical men when called upon to certify in cases of alleged lunacy. In many cases, the insanity is so obviously manifested, that no inquiry apart from the observation of the medical practitioner is necessary to establish its existence; but our legal friends may think differently, and consider that the medical man should not trust to his unaided intelligence, and certify, even when the lunacy is, according to his judgment, palpably portrayed. We much fear that we shall never live to see these halcyon days, that happy medico-legal epoch, when uniformity of opinion shall prevail between the legal and medical mind on questions of insanity. Medical psychologists are gravely told that their "theories" are "crotchety" and "dangerous," and the highest judge in the land, still further clenching the nail, affirms that lunacy is a *fact*, not a *disease*. What can we poor doctors (" mad doctors" if you please, gentle reader), say in opposition to this wonderful exposition of truth? Alas! for how many hundred years has our profession been in a state of gross and blind ignorance as to the true nature and character of disorders of the mind! What we have been treating as a disease of the brain, as a pathological condition, is no disease at all; it is only a "fact," exclaims the oracle of the House of Lords! One of these days we must, to use a parliamentary phrase, propose a conference, and ascertain whether it is not possible to establish a healthier state of feeling between law and medicine, and thus prevent much of that unhappy conflict carried on in our courts of judicature between the members of the two learned professions. Until that happy period arrives we must, I presume, bear with philosophical equanimity the onslaughts to which one particular section of the profession is so often exposed.

The late Sydney Smith declared that the Government would not be alive to the necessity of an improved railroad management until the lives of a few directors or bishops were sacrificed to the gross negligence of railway officials. We much fear that there are some of our legal friends who will persist in ignoring the necessity for confinement in cases of mischievous insanity, until a few of the more distinguished Q.C.s or learned serjeants-at-law are placed *hors de combat* by dangerous and unrestrained lunatics.

